# Prevalence of signs and symptoms in posterior circulation ischemic stroke: a systematic review and meta-analysis

**DOI:** 10.1007/s11739-026-04313-1

**Published:** 2026-03-04

**Authors:** Susanna Sammali, Danilo Caimano, Anna Poggesi, Simone Vanni, Claudio Baracchini, Fana Alemseged, Francesco Arba

**Affiliations:** 1 University of Florence, Florence, Italy; 2 Stroke Unit, Careggi University Hospital, Florence, Italy; 3 Neurology Unit, Cerebrovascular and Neurodegenerative Disease Area of the Department of Medical Specialties, Central Tuscany Local Health Authority, Florence, Italy; 4 Emergency Medicine, University of Florence, Florence, Italy; 5 Stroke Center, Padua University Hospital, University of Padua, Padua, Italy; 6 Department of Medicine and Neurology, Royal Melbourne Hospital, University of Melbourne, Parkville, Australia

**Keywords:** Posterior ischemic stroke, Ischemic stroke, Clinical presentation, Systematic review, Meta-analysis

## Abstract

Posterior circulation (PC) ischemic stroke accounts for approximately a fourth of all acute ischemic strokes and is associated with high disability and mortality. Rapid diagnosis and management can be challenging as it often presents with non-specific signs and symptoms. We performed a systematic review and meta-analysis to describe the prevalence of signs and symptoms in PC ischemic stroke. Two databases (PubMed and EMBASE) were searched for English-written observational studies reporting data on signs and symptoms in patients with a diagnosis of PC ischemic stroke. Data were extracted by two independent researchers. We assessed the risk of bias (RoB) and calculated the pooled prevalence and 95% confidence intervals (CI) using random effect models. We performed subgroup analyses according to type of diagnosis (clinical vs imaging) and location of lesion (cerebellar vs other) in each study. Twenty-eight studies involving three thousand four hundred sixty-two patients were included in the study. RoB was mainly medium/low; in 18/28 studies, diagnosis was performed on imaging. The most common signs were limb weakness (48%; 95% CI 39–58%) and dysarthria (48%; 95% CI 37–59%), while the least frequent was hemianopia (15%; 95% CI 7–25%). The most common symptoms were vertigo/dizziness (41%; 95% CI 30–52%) and nausea/vomiting (41%; 95% CI 32–50%), while the least frequent was diplopia (9%; 95% CI 6–13%). In patients with cerebellar stroke, the most frequent sign was gait ataxia (70%; 95% CI 56–82%), and the most common symptom was vertigo (50%; 95% CI 32–68%). In acute patients with PC ischemic stroke, gait ataxia, vertigo/dizziness, limb weakness, and dysarthria were the most frequent presentations. Our results may inform clinicians and help to refine stroke scales, improving early recognition of PC ischemic stroke in the acute setting.

**Supplementary Information:**

The online version contains supplementary material available at 10.1007/s11739-026-04313-1.

## Introduction

Posterior circulation (PC) ischemic stroke accounts for up to a quarter of all ischemic strokes and is associated with high stroke recurrence, disability, and mortality [1–5].

Compared with stroke in the anterior circulation, the access to reperfusion therapy is frequently limited by longer times from onset to hospital arrival, mainly due to misdiagnosis [6]. Many studies demonstrated a high risk of misidentification of neurological conditions in the emergency department, which can occur in over one-third of patients. Notably, this risk is doubled in PC ischemic stroke compared to anterior stroke due to the nature of its clinical presentation [7–9]. Stroke mimics as well increase the risk of misdiagnoses [10, 11].

Clinical presentations of PC ischemic stroke are highly variable in type and severity, ranging from dizziness, visual field deficits to consciousness disturbances [12]. Furthermore, the National Institutes of Health Stroke Scale (NIHSS) is weighted towards symptoms and signs in the anterior circulation; common clinical presentations of the posterior circulation are not tested, with a risk of underestimating stroke severity and withholding therapy [2, 13, 14]. Consequently, PC ischemic stroke patients with low NIHSS scores have more frequently disability at 3 months than anterior circulation stroke patients [14]. Specific PC stroke scores have been proposed to address these clinical issues, and studies in diverse clinical settings have tried to identify the most prevalent clinical presentation of posterior strokes, with conflicting results. However, such scores have not yet become part of routine clinical evaluation [15–17]. Identification of most frequent signs and symptoms of PC ischemic stroke may be of interest to address the issues in the assessment of these scores.

To provide summary estimates of presenting signs and symptoms of PC ischemic stroke, we performed a systematic review and meta-analysis.

## Materials and methods

### Study search strategy

This study was conducted according to the Preferred Reporting Items for Systematic Reviews and Meta-Analyses guidelines [18]. Data search, extraction, analysis, and interpretation were performed following a pre-specified study protocol developed by the investigators (not registered or published). The literature search was performed using two medical electronic databases (PubMed and EMBASE). There were no time limitations in the bibliographic research regarding the past, while the most recent limit was identified as the year 2024.

### Selection criteria

We considered the studies with the following characteristics as eligible: 1) patients with diagnosis of PC ischemic stroke; 2) at least 20 patients included in the analysis; 3) English-written articles; 4) reporting of clinical presentation (signs/symptoms). We used RAYYAN Software to remove duplicates. Abstracts, case reports, conference presentations, reviews, editorials, and expert opinions were also excluded. Two authors (SS and DC) evaluated the articles independently; a third experienced author (FA) resolved the conflicts. The selected studies were double-checked by all assessors to ensure a common agreement about article selection.

### Data extraction and criteria appraisal

Two independent authors (DC and SS) independently extracted raw data from each included article. The following data were extracted: 1) year of the study; 2) study design and duration; 3) study country; 4) sample size; 5) demographic data of patients (mean age); 6) stroke etiology; 7) presence of vascular occlusion; 8) infarct location; 9) imaging technique used to confirm clinical diagnosis. Raw data were extracted and summarized. The reference lists of all included articles were reviewed to identify further potentially relevant studies. All symptoms and signs mentioned by at least one article were extracted. Following extraction, symptoms and signs that were described slightly differently (e.g., dizziness vs. vertigo) across articles were unified for clarity (e.g., vertigo/dizziness). Discrepancies among signs and symptoms adjudication between the two reviewers were resolved by discussion and consensus with a third author (FA). Eventually, we extracted raw pooled data on a total of nine signs (limb weakness, altered level of consciousness, dysarthria, nystagmus, facial nerve palsy, gait ataxia, hemianopia, dysphagia, limb ataxia) and five symptoms (vertigo, nausea/vomiting, hypoesthesia, headache, diplopia).

### Risk of bias and quality assessment

Risk of bias and quality assessment of all included clinical studies were based on an adaptation of a pre-existing tool (see Supplementary materials) [19]. The assessment was performed independently by two authors (SS and DC). Any discrepancy was solved by consensus. The articles were considered as having a high, moderate, or low risk of bias.

### Statistical analysis

Data were pooled in the meta-analysis when at least three studies had available data on prevalence of any sign/symptom. In all analyses, we used a Generic Inverse Variance approach on Freeman–Tukey double arcsine transformed proportions with a random effects model with DerSimonian–Laird weights. The pooled prevalence and corresponding 95% confidence intervals (CIs) were provided. Statistical heterogeneity was assessed with *I*^2^ statistics and visual inspection of forest plots. Values of ≤ 25%, 25% to 50%, and ≥ 50% were defined as low, moderate, and high degrees of heterogeneity, respectively. Publication bias was assessed by visual inspection of funnel plots and using Egger’s test. All the analyses were performed in February 2025 using the meta-analysis software Stata 18.0.

## Results

**Fig. 1:**
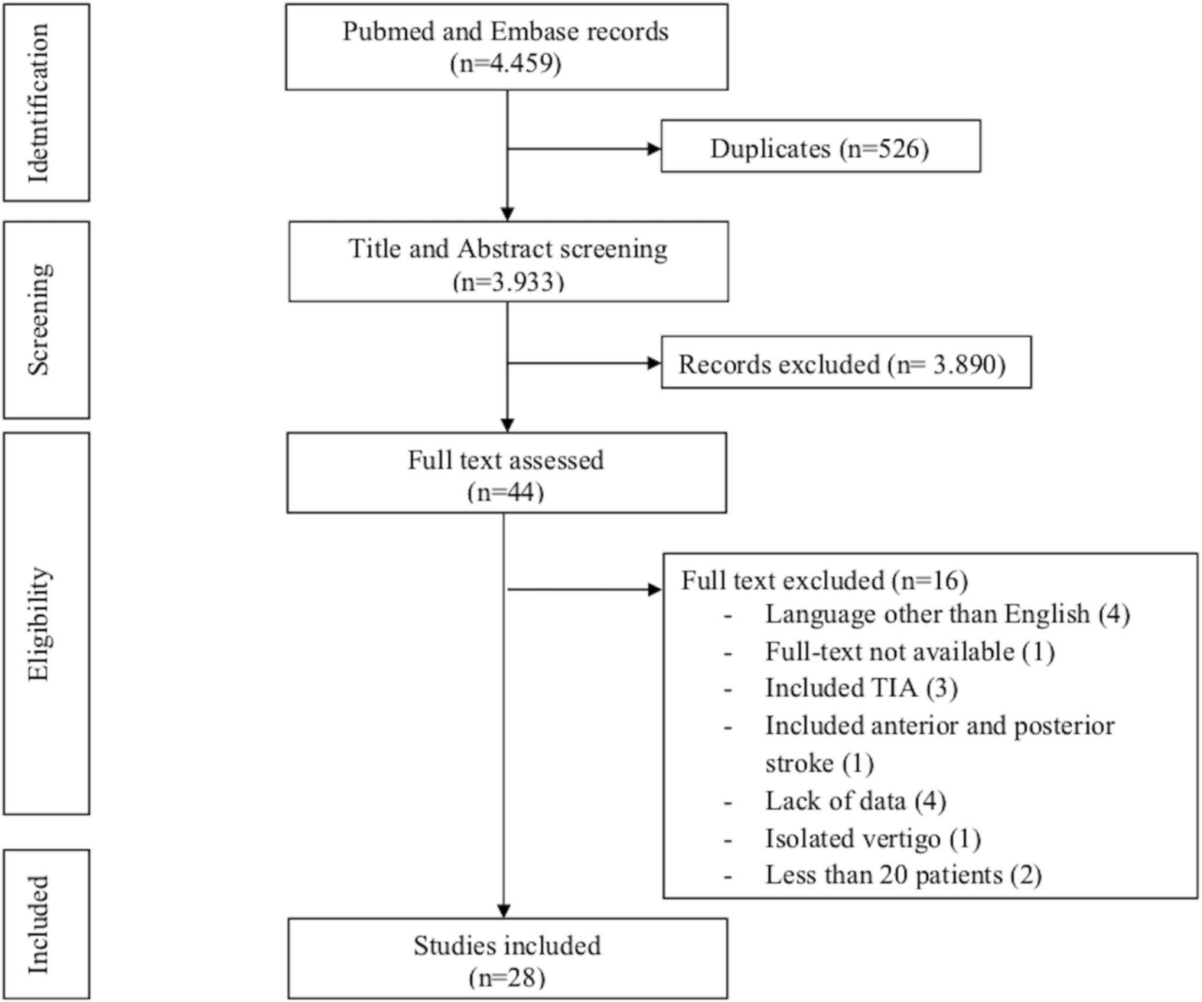
Flowchart of study selection

The initial study search resulted in 4459 studies. After removing duplicates (*N* = 526) and title and abstract screening (*N* = 3890), we selected 44 articles for full-text reading. We excluded 16 articles for topic differences (*n* = 5), language other than English (*n* = 4), full-text unavailable (*n* = 1), and insufficient data or small sample size (*n* = 6), leaving 28 studies and 3462 patients for the final analysis. The study selection is illustrated in Fig. 1.

The general characteristics of the included studies are summarized in Supplemental Table 1. All studies were observational; 20 had a retrospective design [6, 17, 20–37], 6 were prospective [38–43], and 2 were both retrospective and prospective [44, 45]. Included studies were published between 1990 and 2023 and were from Europe (4 studies) [20, 27, 34, 37], America (2 studies) [6, 21], both America and Europe (1 study) [29], Asia (17 studies) [22–26, 28, 31–33, 35, 36, 38–41, 44, 45], Africa (2 studies) [30, 42], Australia (1 study) [43], and Europe, America and Australia (1 study) [17]. Ten studies had a low risk of bias [21, 23, 24, 27, 38, 40, 42–45], sixteen had a medium risk of bias [6, 17, 20, 22, 25, 26, 28–31, 33–37, 41], and two studies had a high risk of bias [32, 39] (Supplemental Fig. 1). Patients were classified as having a PC ischemic stroke on the basis of clinical findings in 9 studies [6, 17, 21, 24, 25, 29, 34, 40, 45], imaging findings in 18 studies [20, 22, 23, 26–28, 30–33, 35–39, 41–44], and both in 1 study [37]. The following diagnostic techniques were applied as reference: cerebral angiogram in one study [20], computed tomography (CT) alone in one report [34], and magnetic resonance (MR) in the remaining studies.

Mean age ranged between 44 and 69 years old, sample size ranged from 30 to 604 patients. Stroke etiology assessment was present in 19 studies (2621 patients) [17, 22–26, 28, 29, 31–33, 35, 36, 38, 39, 41, 43–45] and was as follows: cardioembolic in 325 (12%) patients, atherosclerotic in 820 (31%), small vessel occlusion in 490 (19%), other causes (e.g., dissection) in 350 (13%), unknown/cryptogenic in 643 patients (25%). Fourteen studies (*N* = 644 patients) provided information on vascular occlusion [20–22, 24–26, 28, 29, 31, 32, 35, 38, 43], which ranged from 0 to 100%. Seventeen articles (*N* = 1583 patients) reported specific information on infarct location (2062 patients) [21–29, 31–33, 38–42], which was as follows: 11% medulla (*n* = 236), 9% pons (*n* = 187), 2% midbrain (*n* = 40), 2% brainstem (otherwise not specified) (*n* = 48), and 33% cerebellum (*n* = 658); both brainstem and cerebellum in 33% of patients (*n* = 684), 10% (*n* = 207) in otherwise not specified posterior territory (*n* = 207).

**Fig. 2:**
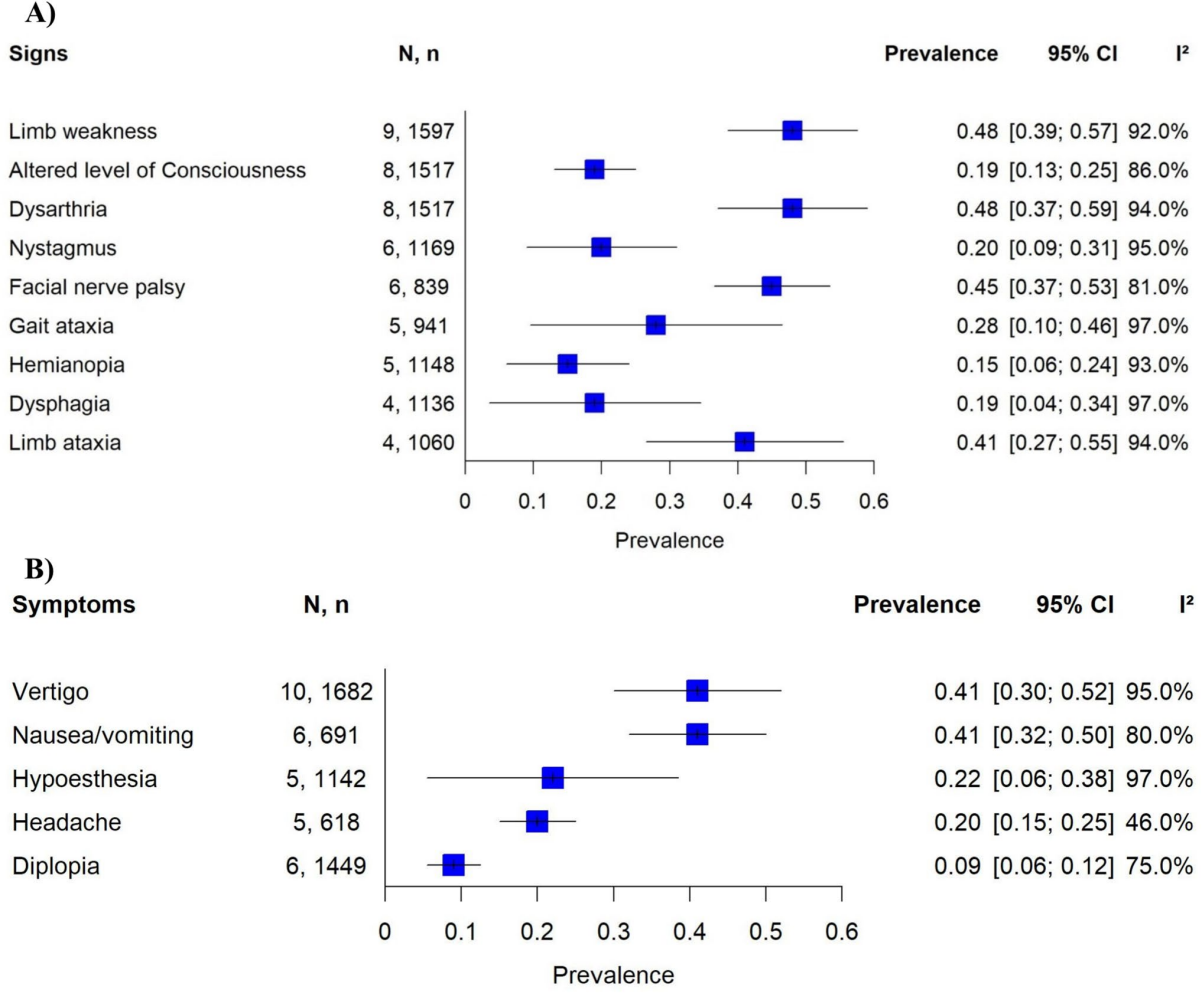
Prevalence of **A** signs and **B** symptoms (cumulative prevalence is shown in forest plots) in posterior circulation ischemic stroke

The pooled prevalence of presenting signs and symptoms is shown in Fig. 2A and B (see Supplemental Figs. 2 and 3 for details). The most frequent signs of presentation were limb weakness (48%; 95% CI 39–58%) and dysarthria (48%; 95% CI 37–59%), while the least frequent was hemianopia (15%; 95% CI 7–25%). Heterogeneity was high across all reports. The most frequent symptoms were vertigo/dizziness (41%; 95% CI 30–52%) and nausea/vomiting (41%; 95% CI 32–50%), whereas diplopia was the least frequent symptom (9%; 95% CI 6–13%). Similar to signs analyses, heterogeneity was high, except for the symptom headache where it was moderate (*I*^2^ = 46%).

### Subgroup analysis: clinical vs imaging

Regarding the diagnostic approach (clinical vs imaging-based diagnosis), dysarthria and vertigo/dizziness were more frequent in studies where stroke diagnosis was confirmed by imaging (for dysarthria, clinical 48%; 95% CI 22–74% vs imaging 54%; 95% CI 43–64%; for vertigo/dizziness, clinical 28%; 95% CI 12–46% vs imaging 51%; 95% CI 39–62%).

For clinical diagnosis alone, the prevalence of diplopia and facial nerve palsy was similar to the main analysis, whereas nausea/vomiting was slightly lower (clinical 35%; 95% CI 30–39% vs 41%; 95% CI 32–50% in the main analysis). For imaging-based diagnosis, the prevalence of dysphagia, limb ataxia, nystagmus, altered level of consciousness, and limb ataxia was comparable to the main analysis.

### Subgroup analysis: cerebellar stroke

In patients with cerebellar stroke, the most frequent sign of presentation was gait ataxia (70%; 95% CI 56–82%), while the least frequent was dysarthria (30%; 95% CI 13–49%), which resulted, respectively, in higher (28%; 95% CI 11–48%) and lower (48%; 95% CI 37–59%) rates than those reported in the main analysis. Regarding symptoms, the most common was vertigo/dizziness (50%; 95% CI 32–68%) and the least frequent was headache (36%; 95% CI 22–52%), which were, respectively, lower (41%; 95% CI 30–52%) and higher (20%; 95% CI 15–25%) in the main analysis (see Supplemental Table 2).

Funnel plots were created for each category and suggested the presence of publication bias. However, Egger’s test did not show any probability of publication bias.

## Discussion

In patients with PC ischemic stroke, we analyzed the pooled prevalence of signs and symptoms and found that limb weakness and dysarthria were the most frequent signs of presentation, whereas vertigo/dizziness and nausea/vomiting were the most common symptoms. On the other hand, hemianopia and diplopia were the least frequent clinical manifestations of PC ischemic stroke. In patients with isolated cerebellar stroke, the most frequent presentations were gait ataxia and vertigo/dizziness.

Clinical presentation of PC ischemic stroke poses relevant diagnostic challenges to clinicians. It has been shown that PC ischemic stroke has lower chances of being adequately treated, given that only 20% of such patients receive reperfusion therapies due to delays and misdiagnosis [46]. A systematic review reported that the most common misdiagnosed clinical presentations of stroke in any localization were nausea/vomiting, vertigo/dizziness, and visual impairment, which are common clinical presentations in PC stroke [47]. Moreover, the lower specificity of symptoms and signs in PC ischemic stroke represents a critical issue in the emergency department and in hyperacute stroke management, and NIHSS has a suboptimal diagnostic accuracy to evaluate PC ischemic stroke severity [48, 49]. Of note, we found that only two (limb weakness, dysarthria) out of four more common clinical presentations are represented in the NIHSS score. Therefore, awareness and information about common symptoms and signs might help with the identification of PC ischemic stroke.

We found that limb weakness, together with dysarthria, was the most frequent sign of presentation, having been reported in almost half of cases, in line with a previous study [50]. Although we observed that up to half of the patients with PC ischemic stroke can present with dysarthria, this presentation can be present in up to 70% of all stroke patients [51, 52]. We observed a relatively lower frequency of hemianopia compared to previous studies which reported a prevalence of around 30% in patients with occlusion of the posterior cerebral artery [53]. This may be due to the inclusion of studies with patients with stroke in locations other than the occipital lobes. In line with previous reports, we found that gait ataxia and nystagmus were more frequent in cerebellar ischemic stroke [54]. On the other hand, the prevalence of limb ataxia was the same between patients with cerebellar stroke and those in the general stroke population, confirming that gait ataxia and nystagmus are highly suggestive signs of cerebellar involvement, whereas limb ataxia is not. Although frequent in PC ischemic stroke, gait ataxia is not routinely assessed in the NIHSS; we therefore advocate systematic assessment of gait and truncal ataxia, particularly when there is suspicion of PC ischemic stroke. In this regard, scales such as post-NIHSS [17] with assessment of truncal ataxia may be useful in the clinical evaluation of patients with suspected PC ischemic stroke.

Regarding symptoms, we observed a low prevalence of diplopia, which was present in nearly one out of ten patients and mainly in studies with only clinical diagnosis of PC ischemic stroke. We did not have information regarding diplopia and stroke confirmed by imaging due to the paucity of studies specifically reporting diplopia in imaging-confirmed PC ischemic stroke. A previous study demonstrated that non-specific terms such as blurred vision were more likely stroke mimics, suggesting the need of a careful examination of associated symptoms/signs to increase diagnostic accuracy [55]. Again, vertigo/dizziness is a challenging clinical presentation, being responsible for nearly one out of ten emergency department admissions and around 20% of neurological referrals in emergency department [56]. A recent systematic review reported that all missed stroke or transient ischemic attack cases in the emergency department had vertigo/dizziness, while false positives (peripheric syndromes diagnosed with stroke) were less than 10% [57]. We found that vertigo/dizziness represented the most frequent symptom in patients with cerebellar stroke, although it was present also in a relevant proportion of all PC ischemic stroke patients. Early recognition of cerebellar stroke is critical to prevent life-threatening events such as cerebellar herniation, and careful examination with simple clinical tools of patients with vertigo/dizziness may help to identify such patients [29, 57–59]. We did not examine signs/symptoms associated with vertigo/dizziness given extraction issues from index studies; however, we acknowledge that other symptoms in combination with vertigo/dizziness might be useful to identify cerebellar strokes.

We analyzed the prevalence of single signs and symptoms but were unable to assess the associations with other concurrent clinical presentations, which could be of value in clinical practice. Our results may be informative for clinicians and researchers that aim to develop diagnostic algorithms for fast triage and identification of suspected PC stroke, providing estimates of clinical presentation from less to more common signs/symptoms. In fact, it has been shown that diagnostic algorithms combining signs and symptoms might increase the diagnostic accuracy for stroke [57, 59], and a dedicated neurological triage may improve in-hospital pathways of patients with neurological symptoms, supporting the use of clinical tools with combined sings/symptoms [60]. To increase sensitivity, such algorithms may incorporate common clinical presentations, whereas to increase specificity, they should incorporate less common clinical presentations. Our subgroup analysis revealed that the prevalence of some signs/symptoms (altered consciousness, dysarthria, and limb weakness) had the same prevalence in studies with clinical diagnosis and imaging diagnosis of PC ischemic stroke, reinforcing the concept that stroke remains a clinical rather than an imaging diagnosis [62]. Of note, diagnostic errors with MR brain occur five times more often in PC than anterior circulation stroke [48, 61], highlighting the value of an accurate clinical examination.

Our study has limitations. First, the retrospective design for the majority of the included studies may affect the overall quality of our results. Nonetheless, all but two studies exhibited a low to moderate risk of bias. Our analysis revealed high statistical heterogeneity for each sign and symptom tested, likely due to the different designs, different inclusion criteria, geographical regions and diagnostic assessments of index studies, suggesting caution in interpreting results. Also, differences in stroke etiology may partly explain the high statistical heterogeneity we found. Moreover, publication bias and small study effect bias may lead to some degree of distortion of our prevalence estimates. Taken together, although our results arise from a systematic review and meta-analysis, the presence of both clinical and methodological high heterogeneity for almost all the clinical presentations evaluated suggests a downgrading of the grade of evidence of our analysis. However, we felt that our results could represent a good trade-off between summarizing existing data and being prone to risk of bias, and suggest the need for further studies specifically targeted on revealing the prevalence of subtle signs of presentation in PC ischemic stroke. Another limitation is the lack of data regarding isolated clinical presentations and more specific signs and symptoms (e.g., bulbar signs such as palatal paralysis or abnormal cough), which hindered our ability to conduct useful analyses in this regard. Again, it should be noted that the frequency of a common stroke etiology such as cardioembolism was different from that previously reported, likely reflecting different diagnostic protocols and patient selection [63]. We also acknowledge that for analysis purposes, we grouped clinical presentations such as nausea/vomiting and vertigo/dizziness, likely introducing imprecision towards higher values on estimate of prevalence. Finally, our protocol has not been previously registered in any systematic review and meta-analysis dataset. The strengths of our study are represented by a systematic approach, the inclusion of studies with clinical and imaging diagnosis of stroke and a summary of clinical presentation of PC ischemic stroke.

In conclusion, in patients with PC ischemic stroke, we found that the most common signs were gait ataxia, limb weakness, and dysarthria, and the most common symptoms were vertigo/dizziness and nausea/vomiting. Awareness about such clinical presentations is of paramount importance to improve the diagnostic accuracy of PC ischemic stroke in the emergency setting early after presentation. Also, the heterogeneous presentations emphasize the need for standardized reporting and further research to refine our understanding, recognition, and evaluation of PC ischemic stroke.

## Supplementary Information

Below is the link to the electronic supplementary material.

Supplementary file1 (DOCX 5435 KB)

## Data Availability

This study is based on previously published data, which are publicly available in the databases and sources cited in the manuscript.

## References

[1] Tao WD, Liu M, Fisher M et al (2012) Posterior versus anterior circulation infarction: how different are the neurological deficits? Stroke 43:2060–2065 22678088 10.1161/STROKEAHA.112.652420

[2] Caplan L (2000) Posterior circulation ischemia: then, now, and tomorrow The Thomas Willis Lecture—2000. Stroke 31:2011–2023 10926972 10.1161/01.str.31.8.2011

[3] Jovin TG, Li C, Wu L et al (2022) Trial of thrombectomy 6 to 24 hours after stroke due to basilar-artery occlusion. N Engl J Med 387:1373–1384 36239645 10.1056/NEJMoa2207576

[4] Tao C, Nogueira RG, Zhu Y et al (2022) Trial of endovascular treatment of acute basilar-artery occlusion. N Engl J Med 387:1361–1372 36239644 10.1056/NEJMoa2206317

[5] Schulz UG, Fischer U (2017) Posterior circulation cerebrovascular syndromes: diagnosis and management. J Neurol Neurosurg Psychiatry 88:45–53 27071644 10.1136/jnnp-2015-311299

[6] Sarraj A, Medrek S, Albright K et al (2015) Posterior circulation stroke is associated with prolonged door-to-needle time. Int J Stroke 10:672–678 23521891 10.1111/j.1747-4949.2012.00952.x

[7] Moeller JJ, Kurniawan J, Gubitz GJ, Ross JA, Bhan V (2008) Diagnostic accuracy of neurological problems in the emergency department. Can J Neurol Sci 35:335–341 18714802 10.1017/s0317167100008921

[8] Scott PA, Silbergleit R (2003) Misdiagnosis of stroke in tissue plasminogen activator-treated patients: characteristics and outcomes. Ann Emerg Med 42:611–618 14581912 10.1016/s0196-0644(03)00443-8

[9] Arch AE, Weisman DC, Coca S et al (2016) Missed ischemic stroke diagnosis in the emergency department by emergency medicine and neurology services. Stroke 47:668–673 26846858 10.1161/STROKEAHA.115.010613

[10] Go S (2015) Posterior circulation ischemic stroke. Emerg Med 112:192–196 PMC6170115 26168589

[11] Tarnutzer AA, Lee SH, Robinson KA et al (2017) ED misdiagnosis of cerebrovascular events in the era of modern neuroimaging. A meta-analysis. Neurology 88:1468–1477 28356464 10.1212/WNL.0000000000003814 PMC5386439

[12] Merwick A, Werring D (2014) Posterior circulation ischaemic stroke. BMJ 348:g3175 24842277 10.1136/bmj.g3175

[13] Inoa V, Aron AW, Staff I, Fortunato G, Sansing LH (2014) Lower NIH Stroke Scale scores are required to accurately predict a good prognosis in posterior circulation stroke. Cerebrovasc Dis 37:251–255 24686370 10.1159/000358869 PMC4956480

[14] Kim JT, Park MS, Choi KH et al (2017) Clinical outcomes of posterior versus anterior circulation infarction with low National Institutes of Health Stroke Scale scores. Stroke 48:55–62 27856952 10.1161/STROKEAHA.116.013432

[15] Olivato S, Nizzoli S, Cavazzuti M et al (2016) e-NIHSS: an expanded National Institutes of Health Stroke Scale weighted for anterior and posterior circulation strokes. J Stroke Cerebrovasc Dis 25:2953–2957 27693107 10.1016/j.jstrokecerebrovasdis.2016.08.011

[16] Gur AY, Lampl Y, Gross B et al (2007) A new scale for assessing patients with vertebrobasilar stroke—the Israeli Vertebrobasilar Stroke Scale (IVBSS): inter-rater reliability and concurrent validity. Clin Neurol Neurosurg 109:317–322 17254701 10.1016/j.clineuro.2006.12.008

[17] Alemseged F, Rocco A, Arba F et al (2022) Posterior National Institutes of Health Stroke Scale improves prognostic accuracy in posterior circulation stroke. Stroke 53:1247–1255 34905944 10.1161/STROKEAHA.120.034019

[18] Moher D, Liberati A, Tetzlaff J, Altman DG et al (2009) Preferred reporting items for systematic reviews and meta-analyses: the PRISMA statement. PLoS Med 6:e1000097 19621072 10.1371/journal.pmed.1000097 PMC2707599

[19] Hoy D, Brooks P, Woolf A et al (2012) Assessing risk of bias in prevalence studies: modification of an existing tool and evidence of interrater agreement. J Clin Epidemiol 65:934–939 22742910 10.1016/j.jclinepi.2011.11.014

[20] Ferbert A, Bruckmann H, Drummen R (1990) Clinical features of proven basilar artery occlusion. Stroke 21:1135–1142 2389292 10.1161/01.str.21.8.1135

[21] Sacco R, Freddo L, Bello JA et al (1993) Wallenberg’s lateral medullary syndrome Clinical-magnetic Resonance Imaging correlations. JAMA Neurol 50:609–614 10.1001/archneur.1993.00540060049016 8503798

[22] Kim JS, Lee JH, Im JH, Lee MC (1995) Syndromes of pontine base infarction. A clinical-radiological correlation study. Stroke 26:950–955 7762044 10.1161/01.str.26.6.950

[23] Kumral E, Bayülkem G, Evyapan D (2002) Clinical spectrum of pontine infarction clinical-MRI correlations. J Neurol 249:1659–1670 12529787 10.1007/s00415-002-0879-x

[24] Kim JS (2003) Pure lateral medullary infarction: clinical± radiological correlation of 130 acute, consecutive patients. Brain 126:1864–1872 12805095 10.1093/brain/awg169

[25] Kim JS (2004) Internuclear ophthalmoplegia as an isolated or predominant symptom of brainstem infarction. Neurology 62:1491–1496 15136670 10.1212/01.wnl.0000123093.37069.6d

[26] Kim JS, Kim J (2005) Pure midbrain infarction clinical, radiologic, and pathophysiologic findings. Neurology 64:1227–1232 15824351 10.1212/01.WNL.0000156520.46056.6B

[27] Deluca C, Moretto G, Di Matteo A et al (2011) Ataxia in posterior circulation stroke: clinical–MRI correlations. J Neurol Sci 300:39–46 21035147 10.1016/j.jns.2010.10.005

[28] Kim SH, Lee JY, Kim DH et al (2013) Factors related to the initial stroke severity of posterior circulation ischemic stroke. Cerebrovasc Dis 36:62–68 23921172 10.1159/000351512

[29] Kase CS, Norrving B, Levine SR et al (1993) Cerebellar infarction. Clinical and anatomic observations in 66 cases. Stroke 24:76–83 8418555 10.1161/01.str.24.1.76

[30] Owolabi LF, Ibrahim A, Musa I (2016) Infratentorial posterior circulation stroke in a Nigerian population: clinical characteristics, risk factors, and predictors of outcome. J Neurosc Rural Pract 7:72–76 10.4103/0976-3147.165427 PMC4750346 26933349

[31] Tohgi H, Takahashi S, Chiba K et al (1993) Cerebellar infarction. Clinical and neuroimaging analysis in 293 patients. Stroke 24:1697–1701 8236346 10.1161/01.str.24.11.1697

[32] Inamasu J, Nakae S, Kato Y, Hirose Y (2018) Clinical characteristics of cerebellar infarction due to arterial dissection. Asian J Neurosurg 13:995–1000 30459855 10.4103/ajns.AJNS_373_16 PMC6208259

[33] Al Hashmi A, Aaron S, Al Sinani A, Pancharatnam D (2021) Clinical and radiological profile of patients presenting with isolated acute cerebellar infarct: a single tertiary center 3 years retrospective study from Central Stroke Unit, Muscat, Oman. J Stroke Med 4:25166085211038210

[34] Halúsková S, Herzig R, Krajícˇková D et al (2021) Acute management should be optimized in patients with less specific stroke symptoms: findings from a retrospective observational study. J Clin Med 10:1143 33803204 10.3390/jcm10051143 PMC7963148

[35] Kim M, Park SY, Lee SE et al (2022) Significance of vertigo, imbalance, and other minor symptoms in hyperacute treatment of posterior circulation stroke. Front Neurol 13:845707 35651338 10.3389/fneur.2022.845707 PMC9150563

[36] Yu C, Zhu Z, Li S et al (2022) Clinical and radiological features of medullary infarction caused by spontaneous vertebral artery dissection. Stroke Vasc Neurol 7:e001180 10.1136/svn-2021-001180 PMC9240456 35241630

[37] Saro-Buendìa M, Torres-Garcìa L, Angel NJ et al (2023) Dizziness evaluation and characterisation of patients with posterior circulation stroke in the emergency department; a case series study. Arch Acad Emerg Med 11:e12 36620730 10.22037/aaem.v11i1.1764 PMC9807946

[38] Akhtar N, Kamran SI, Deleu D et al (2009) Ischaemic posterior circulation stroke in State of Qatar. Eur J Neurol 16:1004–1009 19538206 10.1111/j.1468-1331.2009.02709.x

[39] Mehndiratta M, Pandey S, Nayak R, Alam A (2012) Posterior circulation ischemic stroke—clinical characteristics, risk factors, and subtypes in a North Indian population: a prospective study. Neurohospitalist 2:46–50 23983863 10.1177/1941874412438902 PMC3745183

[40] Tao WD, Liu M, Fisher M et al (2012) Posterior versus anterior circulation infarction: how different are the neurological deficits? Stroke 43:2060–2065 22678088 10.1161/STROKEAHA.112.652420

[41] Chang FC, Yong CS, Huang HC et al (2015) Posterior circulation ischemic stroke caused by arterial dissection: characteristics and predictors of poor outcomes. Cerebrovasc Dis 40:144–150 26228023 10.1159/000437172

[42] El-Sherif M, Esmael A, Elazzouny AA (2016) A comparative clinical study of the characteristics of patients with posterior and anterior circulation ischemic strokes. Egypt J Neurol Psychiatry Neurosurg 53:65–69

[43] Calic Z, Cappelen-Smith C, Cuganesan R et al (2017) Frequency, aetiology, and outcome of small cerebellar infarction. Cerebrovasc Dis 7:173–180 10.1159/000481459 PMC5731170 29130973

[44] Kim JS, Han YS (2009) Medial medullary infarction. Clinical, imaging, and outcome study in 86 consecutive patients. Stroke 40:3221–3225 19628797 10.1161/STROKEAHA.109.559864

[45] Yamada S, Yasui K, Kawakami Y, Hasegawa Y, Katsuno M (2019) DEFENSIVE Stroke Scale: novel diagnostic tool for predicting posterior circulation infarction in the Emergency Department. J Stroke Cerebrovasc Dis 6:1561–1570 10.1016/j.jstrokecerebrovasdis.2019.03.005 30930243

[46] Ekkert A, Milmantiené D, Jokimaityte U, Jatužis D (2023) Posterior circulation stroke patients receive less reperfusion therapy because of late arrival and relative contraindications: a retrospective study. J Clin Med 12:5181 37629223 10.3390/jcm12165181 PMC10455447

[47] Jones SP, Bray JE, Gibson JME et al (2021) Characteristics of patients who had a stroke not initially identified during emergency prehospital assessment: a systematic review. Emerg Med J 38:387–393 33608393 10.1136/emermed-2020-209607 PMC8077214

[48] Hoyer C, Szabo K (2021) Pitfalls in the diagnosis of posterior circulation stroke in the emergency setting. Front Neurol 12:682827 34335448 10.3389/fneur.2021.682827 PMC8317999

[49] Tarnutzer AA, Lee SH, Robinson KA et al (2017) ED misdiagnosis of cerebrovascular events in the era of modern neuroimaging. A meta-analysis. Neurology 88:1468–1477 28356464 10.1212/WNL.0000000000003814 PMC5386439

[50] Hand PJ, Kwan J, Lindley RI, Dennis MS, Wardlaw JM (2006) Distinguishing between stroke and mimic at the bedside: the brain attack study. Stroke 37:769–775 16484610 10.1161/01.STR.0000204041.13466.4c

[51] De Cock E, Oostra K, Bliki L et al (2021) Dysarthria following acute ischemic stroke: prospective evaluation of char- acteristics, type and severity. Int J Lang Commun Disord 0:1–9 10.1111/1460-6984.12607 33580596

[52] Urban PP, Wicht S, Vukurevic G et al (2001) Dysarthria in acute ischemic stroke. Lesion topography, clinicoradiologic correlation, and etiology. Neurology 56:1021–1027 11320172 10.1212/wnl.56.8.1021

[53] Benke T, Dazinger F, Pechlaner R et al (2021) Lesion topography of posterior cerebral artery infarcts. J Neurol Sci 428:117585 34371243 10.1016/j.jns.2021.117585

[54] Wright J, Huang C, Strbian D, Sundarajan S (2014) Diagnosis and management of acute cerebellar infarction. Stroke 45:e56–e58

[55] Helboe KS, Eddelien HS, Kruuse C (2023) Visual symptoms in acute stroke. A systematic review of observational studies. Clin Neurol Neurosurg 229:107749 37163931 10.1016/j.clineuro.2023.107749

[56] Hansen CK, Fisher J, Joyce NR, Edlow JA (2015) A prospective evaluation of indications for neurological consultation in the emergency department. Int J Emerg Med 8:26 26223983 10.1186/s12245-015-0074-3 PMC4514733

[57] Vanni S, Pecci R, Edlow JA et al (2017) Differential diagnosis of vertigo in the emergency department: a prospective validation study of the STANDING algorithm. Front Neurol 8:590 29163350 10.3389/fneur.2017.00590 PMC5682038

[58] Hornig CR, Rust DS, Busse O, JauB M, Laun A (1994) Space-occupying cerebellar infarction clinical course and prognosis. Stroke 25:372–374 8303748 10.1161/01.str.25.2.372

[59] Kattah JC, Talkad AV, Wang DZ et al (2009) HINTS to diagnose stroke in the acute vestibular syndrome: three-step bedside oculomotor examination more sensitive than early MRI diffusion-weighted imaging. Stroke 40:3504–3510 19762709 10.1161/STROKEAHA.109.551234 PMC4593511

[60] Hoyer C, Stein P, Rausch HW et al (2019) The use of a dedicated neurological triage system improves process times and resource utilization: a prospective observational study from an interdisciplinary emergency department. Neurol Res Pract 1:29 33324895 10.1186/s42466-019-0036-y PMC7650056

[61] Edlow BL, Hurwitz S, Edlow JA (2017) Diagnosis of DWI-negative acute ischemic stroke: a meta-analysis. Neurology 89:256–262 28615423 10.1212/WNL.0000000000004120 PMC5513816

[62] Ferretti S, Baldini M, Lombardo I et al (2023) Radiological-but not clinical-stroke mimic in a patient with an impaired state of consciousness. Neurol Sci 44:1441–1443 36427101 10.1007/s10072-022-06515-9 PMC9702691

[63] Caplan LR, Wityk RJ, Glass TA et al (2004) New England Medical Center posterior circulation registry. Ann Neurol 56:389–398 15349866 10.1002/ana.20204

