# Supplementary material for: Prevalence of signs and symptoms in posterior circulation ischemic stroke: a systematic review and meta-analysis
Source: Intern Emerg Med. 2026 Mar 4;21(5):1523–30. doi: 10.1007/s11739-026-04313-1 (PMC13452841; doi:10.1007/s11739-026-04313-1)
Supplement: Supplementary file 1 [file 11739_2026_4313_MOESM1_ESM.docx]

**Supplementary Materials**

**Supplemental Figure 1, Risk of bias assessment** Adapted from Hoy D, Brooks P, Woolf A, Blyth F, March L, Bain C, et al. Assessing risk of bias in prevalence studies: modification of an existing tool and evidence of interrater agreement. J Clin Epidemiol. 2012;65:934-939

**Q_1_:** Was the study target population a close representation of the national population (adults, children, or both) in relation to relevant variables?

**Q_2_:** Was the sampling frame a true or close representation of the target population as suggested by the study title and objectives?

**Q_3_:** Was some form of random selection used to select the sample, OR, was a census undertaken? (census or consecutive/exhaustive sampling)

**Q_4_:** Was the likelihood of non-participation bias minimal? (probability that investigators have failed to include subjects that would normally be eligible)

**Q_5_:** Were data collected prospectively directly from the participants (as opposed to mere review of medical records or retrospective data collection)?

**Q_6_:** Was the process of identifying patients with posterior circulation stroke appropriate and clearly described?

**Q_7_:** Was the diagnostic method (brain imaging) used to identify posterior stroke clearly described?

**Q_8_:** Was the same assessment protocol used for all the participants?

**Q_9_:** Were the signs/symptoms clearly presented? (adequate reporting of data within each category of lesion/patients + discrete categories/no overlapping + no errors requiring a guess/adjustments)

**Q_10_:** Were the numerator(s) and denominator(s) for the calculation of the prevalence of high-risk plaque appropriate?

**Interpretation of the score**

8 – 10: Low Risk of Bias / High-quality study. Further research is very unlikely to change our confidence in the estimate.

5 – 7: Moderate Risk of Bias / Moderate-quality study. Further research is likely to have an important impact on our confidence in the estimate and may change the estimate.

4 or less: High Risk of Bias / Low-quality study. Further research is very likely to have an important impact on our confidence in the estimate and is likely to change the estimate. Further research is mandatory.

L, low risk of bias; M, medium risk of bias; H, high risk of bias.

Altered level of Consciousness

Limb weakness

Dysarthria

Nystagmus

Facial nerve palsy

Gait Ataxia

Hemianopia

Dysphagia

Limb Ataxia

**Supplemental Figure 2.** Prevalence of signs in posterior circulation ischemic stroke.

**Supplemental Figure 3.** Prevalence of symptoms in posterior circulation ischemic stroke.

| **Author,**  **year** | **Country** | **Study design, duration (years)** | **Inclusion criteria** | **Patients**  **(n)** | **Age^#^** | **Stroke etiology** | **Vascular occlusion detection**  **(N (%) studied, n/N (%) with occlusion)** | **Infarct location**  **(%)** | **Imaging technique (%)** |
| --- | --- | --- | --- | --- | --- | --- | --- | --- | --- |
| Ferbert et al., 1990 | Germany | Retrospective, 11 | I: basilar artery occlusion | 85 | NA  (26-75) | NA | 100% | NA | Cerebral angiogram: 100% |
| Sacco et al., 1992 | USA | Retrospective case series, 7 | C: lateral medullary syndrome | 33 | 58 (24-82) | NA | 23 (70%), 15/23 (65%) | - Medulla 36%  - Cerebellum 9%  - Other not specified brainstem areas 55% | CT: 100%  MR: 67% |
| Kim JS et al., 1995 | South Korea | Retrospective, 5 | I: pontine infarct | 37 | 61  (36-85) | - SVD 11 (30%)  - UNK 26 (70%) | 6 (16%), 0/16 (0%) | Pons 100% | CT: 81%  MR: 86% |
| Kumral et al., 2002 | Turkey | Retrospective, 6 | I: pontine infarct | 150 | 63±12 | - LAD 27 (18%)  - CE 12 (8%)  - SVD 90 (60%)  - UNK 16 (11%)  - OTH 5 (3%) | NA | Pons 100% | MR: 100% |
| Kim JS,  2003 | South Korea | Retrospective, 9 | C: lateral medullary infarct | 130 | 58±12 | - LAD 65 (50%)  - CE 7 (5%)  - SVD 17 (13%)  - UNK 20 (17%)  - OTH 21 (15%) | 123 (95%), 52/123 (42%) | Medulla 100% | MR: 100% |
| Kim JS,  2004 | South Korea | Retrospective, 9 | C: internuclear ophthalmoplegia | 30 | 62  (41-82) | - SVD 29 (97%)  - UNK 1 (3%) | 100%, 2/30 (6%) | Brainstem 100% | MR: 100% |
| Kim JS et al., 2005 | South Korea | Retrospective, 8 | I: midbrain infarct | 40 | 65  (47-81) | - LAD: 25 (63%)  - CE 1 (3%)  - SVD: 10 (25%)  - UNK 4 (9%) | 100%, 0/40 (0%) | Midbrain 100% | MR: 100% |
| Akhtar et al., 2009 | Qatar | Prospective observational, 4 | I: posterior circulation ischemic stroke | 116 | 53±16 | - LAD 54 (47%)  - CE 17 (15%)  - SVD 19 (17%)  - UNK 3 (2%)  - OTH 23 (19%) | 100%, 49/116 (42%) | - Proximal territory 40%  - Middle territory 27%  - Multiple territories 23%  - Distal territory 10%* | CT: 100%  MR: 95% |
| Kim JS et al.,  2009 | South Korea | Retrospective and prospective, 13 | I: medial medullary infarct | 86 | 62±10 | - LAD 53 (62%)  - CE 1 (1%)  - SVD 24 (28%)  - UNK 6 (7%)  - OTH 2 (2%) | NA | Medulla 100% | MR: 100% |
| Deluca et al., 2010 | Italy | Retrospective, 2 | I: cerebellar infarct | 92 | 60±15 | NA | NA | Cerebellum 100% | MR: 100% |
| Mehndiratta et al., 2012 | India | Prospective, NA | I: posterior circulation ischemic stroke | 80 | 52±14 | - LAD 61 (76%)  - CE 8 (10%)  - SVD 3 (3%)  - UNK 6 (8%)  - OTH 2 (3%) | NA | - Medulla and proximal cerebellum 30%  - Pons and middle cerebellum 4%  - Midbrain and distal territories 66% § | CT: 100%  MR: NA |
| Tao et al., 2012 | China | Prospective, 6 | C: acute ischemic stroke | 302 | 61±14 | NA | NA | NA | MR: 100% |
| Kim SH et al., 2013 | South Korea | Retrospective, 5 | I: acute posterior circulation ischemic stroke | 604 | 67±11 | - LAD 155 (26%)  - CE 74 (12%)  - SVD 140 (23%)  - UNK 219 (36%)  - OTH 16 (3%) | 100%, 328/604 (54%) | - Medulla and proximal cerebellum 19%  - Pons and middle cerebellum 35%  - Midbrain and distal territories 33% §  - Bilateral or multiple 13% | MR: 100% |
| Sarraj et al., 2013 | USA | Retrospective, 3 | C: acute ischemic stroke | 31 | 65±14 | NA | NA | NA | NA |
| Chang et al., 2015 | Taiwan | Prospective, 3 | I: acute posterior circulation ischemic stroke  E: arterial dissection | 74 | 66±16 | Dissection 74 (100%) | NA | NA | MR: 100% |
| El-Sherif et al., 2016 | Egypt | Prospective, 1 | I: acute ischemic stroke | 60 | 60±14 | NA | NA | NA | MR: 100% |
| Kase et al.,  2016 | USA and Sweden | Retrospective, NA | C: cerebellar infarct | 66 | 61  (18-88) | - LAD 3 (5%)  - CE 25 (38%)  - SVD 20 (30%)  - UNK 18 (27%) | 56 (85%), 11/56 (20%) | Cerebellum 100% | CT or MR: 100% |
| Owolabi et al., 2016 | Nigeria | Retrospective, 7 | I: acute posterior circulation stroke | 38 | NA | NA | NA | NA | CT or MR: 100% |
| Tohgi et al., 2016 | Japan | Retrospective, 5 | I: cerebellar infarct | 293 | 65±12 | - LAD 143 (49%)  - UNK 150 (51%) | 157 (54%), NA | - Cerebellum 100% | CT or MR: 100% |
| Calic et al., 2017 | Australia | Prospective, 5 | I: cerebellar infarct | 108 | 67±13 | - LAD 40 (39%)  - CE 40 (39%)  - UNK 22 (22%) | 90 (83%), NA | Cerebellum 100% | CT: 100%  MR: 85% |
| Inamasu et al., 2018 | Japan | Retrospective, 8 | I: cerebellar infarct | 49 | 63±13 | - LAD 17 (35%)  - CE 18 (37%)  - OTH 14 (28%) | 100%, NA | Cerebellum 100% | CT: 100%  MR: 100% |
| Yamada et al., 2019 | Japan | Retrospective and prospective, 4 | C: acute dizziness, imbalance and/or vertigo | 100 | 69±13 | - LAD 34 (34%)  - CE 11 (11%)  - SVD 28 (28%)  - OTH 27 (27%) | NA | - Lateral medulla 9%  - Other territories 91% | MR: 100% |
| Al Hashmi et al., 2021 | Oman | Retrospective, 3 | I: cerebellar infarct | 48 | 59  (18-90) | - LAD 15 (31%)  - CE 14 (29%)  - SVD 1 (2%)  - UNK 14 (29%)  - OTH 4 (9%) | NA | Cerebellum 100% | CT: 96%  MR: 48% |
| Halùskovà et al., 2021 | Czech Republic | Retrospective observational, 6 | C: acute ischemic stroke | 102 | NA | NA | NA | NA | CT: 100% |
| Alemseged et al., 2022 | Australia, New Zealand, USA, Europe | Retrospective, 14 | C: posterior circulation ischemic stroke; NIHSS score <10 | 267 | 63±16 | - LAD 49 (18%)  - CE 51 (19%)  - SVD 39 (15%)  - UNK 105 (39%)  - OTH 23 (9%) | NA | NA | NA |
| Kim M et al., 2022 | South Korea | Retrospective observational, 5 | I: acute posterior circulation ischemic stroke | 228 | Period 1: 65±14  Period 2: 67±12 | - LAD 79 (35%)  - CE 46 (20%)  - SVD 59 (26%)  - UNK 33 (14%)  - OTH 11 (5%) | 100%, 102/228 (45%) | NA | MR: 100% |
| Yu et al, 2022 | China | Retrospective, 10 | I: medullary infarction caused  E: VA dissection | 128 | Spontaneous VA dissection: 44 (34-53)  Non-spontaneous VA dissection: 58 (52-66) | Dissection 128 (100%) | 100%, NA | NA | CT: 83%  MR: 100% |
| Saro-Buendìa et al., 2023 | Spain | Case series, observational, 4 | C: dizziness  I: posterior circulation ischemic stroke | 85 | 73  (64-79) | NA | NA | NA | MR: 100% |

**Supplemental Table 1**, **Summary of included studies**

AICA = Anterior inferior cerebellar artery; BA = Basilar artery; BAD = Branch atheromatous disease; C = Patients included by clinical presentation; CE = Cardio embolism; CT = Computed tomography; E = Etiology; I = Patients included by imaging characteristics; IQR = Interquartile range; LAD = Large artery disease; MR = Magnetic resonance; NIHSS = National Institute of Health Stroke Scale; OTH = Other; PICA = Posterior inferior cerebellar artery; SCA = Superior cerebellar artery; SVD = Small vessel disease; UNK = Unknown; VA = Vertebral artery.

\# As provided by each study when specified: mean±standard deviation or interquartile range; median±interquartile range.

* No exact definition of the territories was reported.

§ Midbrain, thalamus, distal cerebellum, occipital and medial temporal lobe

| **Sign/Symptom** | **Studies** | **Proportion [95% CI]** |
| --- | --- | --- |
| Dysarthria | Kase, 1993; Deluca, 2010; Calic, 2017; Hashmi, 2021 | 0.30 [0.13-0.49] |
| Gait ataxia | Kase, 1993; Deluca, 2010; Calic, 2017; Inamasu, 2018; Hashmi, 2021 | 0.70 [0.56-0.82] |
| Limb ataxia | Kase, 1993; Deluca, 2010; Calic, 2017; Hashmi, 2021 | 0.44 [0.15-0.76] |
| Nystagmus | Kase, 1993; Deluca, 2010; Calic, 2017; Hashmi, 2021 | 0.47 [0.28-0.67] |
| Headache | Kase, 1993; Calic, 2017; Inamasu, 2018; Hashmi, 2021 | 0.36 [0.22-0.52] |
| Nausea-vomiting | Kase, 1993; Deluca, 2010; Calic, 2017; Inamasu, 2018; Hashmi, 2021 | 0.48 [0.33-0.65] |
| Vertigo | Kase, 1993; Deluca, 2010; Calic, 2017; Inamasu, 2018; Hashmi, 2021 | 0.50 [0.32-0.69] |

**Supplemental Table 2, Cerebellar stroke**

| **Sign/Symptom** | **Studies** | **Proportion**  **[95% CI]** |
| --- | --- | --- |
| Hemianopia | Tao, 2012; Kim SH, 2013; El-Sherif, 2016;  Haluskova, 2021 | 0.14  [0.0.5-0.26] |
| Dysphagia | Kim SH, 2013; Chang, 2015; Alemseged, 2022 | 0.17  [0.03-0.41] |
| Limb weakness | Akhtar, 2009; Tao, 2012; Kim SH, 2013; Sarraj, 2013; Chang, 2015; El-Sherif, 2013; Haluskova, 2021; Kim M, 2022 | 0.49  [0.38-0.59] |
| Headache | Tao, 2012; Chang, 2015; El-Sherif, 2016; Haluskova, 2021 | 0.18  [0.14-0.21] |
| Nausea-vomiting | Akhtar, 2009; Tao, 2012; Sarraj, 2013; El-Sherif, 2016; Haluskova, 2021 | 0.41  [0.30-0.52] |
| Vertigo | Akhtar, 2009; Tao, 2012; Kim SH, 2013; Sarraj, 2013; Chang, 2015; El-Sherif, 2016; Haluskova, 2021, Kim M, 2022; Saro-Buendia, 2023 | 0.39  [0.28-0.51] |

**Supplemental Table 3, Prevalence analysis after the exclusion of studies at high risk of bias.**
